# The Beneficial Effects of Pterostilbene on Post-Thawed Bovine Spermatozoa

**DOI:** 10.3390/ani13172713

**Published:** 2023-08-25

**Authors:** Vasiliki Sapanidou, Maria Tsantarliotou, Sophia Lavrentiadou, Elena Tzekaki, Ioannis Efraimidis, Theodoros Lialiaris, Byron Asimakopoulos

**Affiliations:** 1 Laboratory of Physiology, School of Veterinary Medicine, Faculty of Health Sciences, Aristotle University of Thessaloniki, University Campus, 54124 Thessaloniki, Greece; slavrent@vet.auth.gr (S.L.); ioannisefre@yahoo.gr (I.E.); 2 Department of Agriculture, School of Agricultural Sciences, University of Western Macedonia, 53100 Florina, Greece; 3 Laboratory of Biochemistry, Department of Chemistry, Aristotle University of Thessaloniki, University Campus, 54124 Thessaloniki, Greece; etzekaki@chem.auth.gr; 4 Laboratory of Genetics, Faculty of Medicine, School of Health Science, Democritus University of Thrace, University Campus-Dragana, 68100 Alexandroupolis, Greece; lialiari@med.duth.gr; 5 Laboratory of Physiology, Faculty of Medicine, School of Health Science, Democritus University of Thrace, University Campus-Dragana, 68100 Alexandroupolis, Greece; basima@med.duth.gr

**Keywords:** sperm, frozen, quality of frozen semen, pterostilbene, antioxidants

## Abstract

**Simple Summary:**

The present study was designed to investigate the effect of pterostilbene, a phenolic compound found in fruits and red wine, on bovine sperm quality characteristics. The spermatozoon was safely handled in the laboratory before subjecting it to Assisted Reproductive Techniques, such as in vitro fertilization, which generates free radicals that might negatively affect structural integrity and fertilizing capacity. An approved approach to protect spermatozoa is the supplementation of the media with exogenous antioxidants. The multiple benefits and pleiotropic actions of antioxidants make them scientifically interesting and popular. In this study, the effect of two different concentrations (10 μΜ and 25 μΜ) of pterostilbene have been tested during two incubation periods, 60 min and 240 min, after thawing. The addition of 25 μM pterostilbene in the bovine sperm preparation media had advantageous effects on motility, viability, intracellular superoxide anion concentration, and acrosomal status of spermatozoa. Thus, the role of pterostilbene seems potentially beneficial for spermatozoa and should be further investigated.

**Abstract:**

Reactive Oxygen Species (ROS), primarily produced by cellular metabolism, are highly reactive molecules that modify cellular compounds. During sperm preparation in Assisted Reproductive Techniques (ARTs), intrinsic and extrinsic sources of ROS can impact spermatozoa’s oxidative status. The modification of the media with compounds that enhance sperm quality characteristics is of great significance. The current study investigated the effect of pterostilbene, a phenolic compound, on bovine sperm quality. Cryopreserved spermatozoa from six bulls were thawed, supplemented with pterostilbene (0, 10 μΜ, 25 μΜ) and incubated for 60 min and 240 min. Spermatozoa were analyzed in terms of motility, viability, acrosomal status and intracellular concentration of superoxide anion in each time point. The incubation of spermatozoa with 25 μΜ pterostilbene resulted in the preservation of quality parameters through superoxide anion mitigation, while its presence in capacitating conditions resulted in higher percentage of acrosome-reacted spermatozoa. The results of the present study indicate that the addition of pterostilbene prevents oxidative insult to spermatozoa and preserves the sperm quality parameters.

## 1. Introduction

Like all living cells, spermatozoa use oxygen to oxidate organic molecules and survive; thus, they constantly face the oxygen paradox, in that while oxygen is essential for the metabolic activity, the intermediate byproducts, called Reactive Oxygen Species (ROS), are highly reactive molecules that interact with all cellular compounds [1]. ROS can modify the structure of proteins, lipids, and DNA. They are known inducers of lipid peroxidation (LPO), DNA fragmentation and apoptosis which result in alterations of membrane fusion events, loss of motility, and ATP depletion [1,2]. Spermatozoa are not heavily equipped with antioxidants, so the equilibrium between ROS production and neutralization is maintained by the seminal plasma which is enriched with both enzymatic and non-enzymatic antioxidants [3].

During Assisted Reproductive Techniques (ARTs), sperm handling that aims to harvest viable and motile spermatozoa also leads to the overproduction of ROS and deterioration of sperm quality [4]. ROS are overproduced due to the freeze/thaw process [5] while centrifugation deprives spermatozoa from the exogenous protection of seminal plasma which is rich in antioxidant compounds [3,6], resulting in the exposure of spermatozoa to higher oxygen tension (21%) compared to that of the genital tract (5%) [7]. This disturbance between ROS production and antioxidant activity, called Oxidative Stress, negatively influences fertilizing capacity [1,2,8]. OS-induced low fertilization rates both in vivo and in vitro appear to be very challenging for andrologists. Different antioxidant compounds have been utilized in vitro as additives to extenders of culture media to maintain sperm quality characteristics and ameliorate the fertilizing capacity of spermatozoa [9,10,11]. However, the addition of any substances should be carried out with respect to sperm physiology. Controlled amounts of ROS are important for normal sperm function because ROS act as signaling molecules necessary for sperm maturation, hyperactivation, capacitation and acrosome reaction [4,12,13,14].

Phenolic compounds are ubiquitous phytochemicals that are present in fruits, vegetables, olive oil and red wine [15]. More than 8000 phenolic molecules exist which are important for the growth and reproduction of the plants, providing protection against pathogens and predators [16]. The huge family of polyphenols is divided into two main groups: non-flavonoids and flavonoids. Non-flavonoids include phenolic acids, stilbenes, and lignans [15,16].

**Scheme 1:**
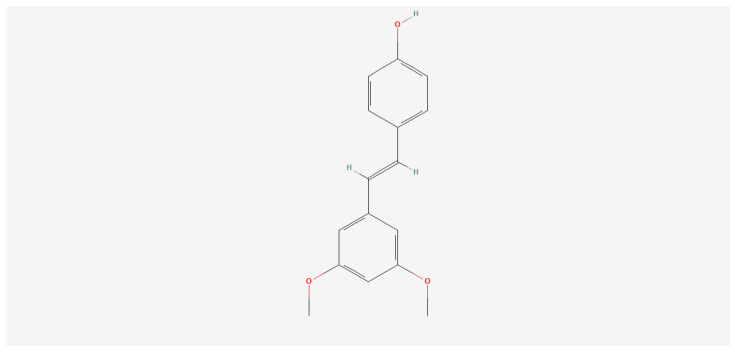
Chemical structure of pterostilbene (National Center for Biotechnology Information. “PubChem Compound Summary for CID 5281727, Pterostilbene” PubChem https://pubchem.ncbi.nlm.nih.gov/compound/Pterostilbene, accessed on 10 August 2023).

Stilbenes are phytoalexins (antibiotics produced by a plant in response to environmental stresses [16]) with pleiotropic functions, such as antioxidant, neuroprotective, and antitumorigenic, which are mainly exerted through chelation of metallic anions and they provide protection from LPO [17,18]. Resveratrol is a non-flavonoid phenolic compound which has garnered attention in recent years [17,19]. However, pterostilbene (PT, trans-3,5-dimethoxy-4-hydroxystilbene), found primarily in blueberries and grape species of *Vitis vinifera,* is another non-flavonoid compound which is considered potentially beneficial [19] because it can be synthesized via biological and chemical methods; additionally, it can be extracted from the natural sources [20]. Due to its chemical structure (Scheme 1), PT is characterized by increased bioavailability (80% bioavailability compared to 20% for resveratrol, which is structurally similar) which renders it very advantageous [21]. Many properties have been attributed to PT, such as antioxidant, anti-inflammatory, and anti-apoptotic [22,23,24]. These actions have been verified in different cell types, such as platelets [25], keratinocytes [26], corneal epithelial cells [27] and bovine embryos [28]. Its antioxidant activity is responsible for the protective effect of PT which is mainly exerted through scavenging of hydrogen peroxide H_2_O_2_ and superoxide anion (O_2_^−^) and the increased expression of catalase, total glutathione, glutathione peroxidase, glutathione reductase, and superoxide dismutase (SOD) in different cell lines [29,30,31]. This effect requires beta estrogen receptor (ERβ), which implies that PT is a ERβ agonist and acts as phytoestrogen, similar to resveratrol [31].

To the best of our knowledge, PT has not been tested on spermatozoa before. It has been employed in culture of embryos, both bovine and murine [28,32]. The outcomes of these studies appear to be too controversial to draw a conclusion. PT (0.25 μM) showed anti-apoptotic and antioxidant capacities and significantly increased the developmental competence of mouse embryos [32]. This effect was not verified in cultured bovine embryos where PT supplementation (0.33 μΜ) resulted in a lower rate of blastocyst production on days 7 and 8 and decreased the percentage of transferable embryos on day 7 due to the elimination of ROS production [28].

The present study was designed to evaluate the effects of PT on the quality of spermatozoa. We hypothesized that supplementation of bovine sperm preparation media with PT would improve sperm motility and viability. Moreover, PT supplementation was tested under capacitating conditions to investigate the effect of PT on sperm capacitation/acrosome reaction.

## 2. Materials and Methods

For the purpose of the present study, three Limousine, two Holstein, and one Brown Swiss bull of proven fertility were selected as sperm donors. The samples were kindly offered by the Center of Artificial Insemination of Thessaloniki (Ionia, Greece). The age of bulls ranged from 48 to 60 months at the beginning of our experiment. The ejaculates were collected with an artificial vagina between November 2022 and February 2023. The authors recognize the potential limitations the low number of bulls may pose in terms of generalizability, but practical, ethical, and technical reasons necessitate that a small number of animals should be used in the study. Pooling of semen from each animal was chosen as a means to compensate for possible individual or breed differences.

After semen collection, the samples should fulfill specific quality criteria in terms of motility, morphology, and viability to be classified as suitable for cryopreservation and artificial insemination (>70% initial motility, >75% viability, and a total concentration of at least 4 × 10^9^ spermatozoa/mL). The ejaculates were cryopreserved with a Tris-egg yolk extender (20% Tris-egg yolk, 7% glycerol, 78 mM citric acid, 69 mM fructose, 50 μg tylosin, 250 μg gentamycin, 150 μg lincomycin, and 300 μg spectinomycin in each mL of extended frozen semen) and packed into 0.5 mL plastic straws, each one containing approximately 50 × 10^6^ spermatozoa/mL. The semen was then cooled to 4 °C for 4 h before cryopreservation. Temperature was decreased in order to reach −110 °C within 10 min. The straws were then plunged into liquid nitrogen (−196 °C). The storage period of semen in liquid nitrogen ranged from 2 to 24 months.

At the beginning of each experiment, six straws were simultaneously thawed via immersion in a water bath (37 °C, 40 s) and combined into a sterile conical tube (CellstarTubes, Greiner Bio One, Frickenhausen, Germany) to form a sperm pool. All assays were conducted 6 times (*n* = 6). All reagents were purchased from Sigma-Aldrich (Merck SA, Athens, Greece), unless otherwise specified. 

### 2.1. PT Preparation

PT of high purity (>99%) was stored as a powder at +4 °C in the dark. The stock solution of PT (20 mM) was prepared in dimethylosulfoxide (DMSO). The working solution of PT (500 μM) was freshly prepared before each experiment via dilution of the stock solution with Sperm Tyrode’s Albumin Lactate Pyruvate (TALP). An equal volume of medium was removed from the tubes before the supplementation of PT. The highest final concentration of DMSO in the media was 0.02%.

### 2.2. Motility and Viability Assessment

Sperm samples were washed twice with 3× volumes of Sperm TALP (100 mM NaCl, 3.1 mM KCl, 25 mM NaHCO3, 0.29 mM NaH_2_PO_4_, 21.6 mM sodium lactate, 2 mM CaCl_2_, 1.5 mM MgCl_2_, 10 mM Hepes sodium salt, supplemented with 1 mM sodium pyruvate and 50 μg/mL gentamycin in water for embryo transfer) and centrifuged at 300× *g* for 10 min (25 °C). Sperm concentration was determined with a haemocytometer (OptikLabor, Grale HDS, New South Wales, Australia). The washed pool of spermatozoa was divided into three tubes and Sperm TALP was added to achieve a concentration of 50 × 10^6^ cells/mL in each tube. One tube served as a control, while the others were supplemented with two different concentrations of PT (10 μM and 25 μM). Τhe motility was assessed via Computer Assisted Sperm Analyzer (CASA), consisting of a triocular optical phase microscope (NiconEclipse C1, Nikon, Tokyo, Japan), a warming plate (Tokai, Tokyo, Japan) at 37 °C and a Baler Scout CCD digital camera (Basler Vision Technologies, Ahrensburg, Germany), which was connected to a computer (Dell, Austin, TX, USA). 

Five μL aliquots of sperm suspension were placed on a pre-warmed glass slide (Citoglas^TM^, Citotest Labware Manufacturing Co.; Haimen, China) and were analyzed using Integrated Semen Analysis System Software (ISAS MvCo, Valencia, Spain) at three different time points (0 min, 60 min, and 240 min). The following parameters were compared between the groups: rapid (%), medium (%), slow (%), static (%), progressive motile (%), curvilinear velocity (VCL, μm/s), straight linear velocity (VSL, μm/s), average path velocity (VAP, μm/s), and average lateral head displacement (ALH, μm). 

The CASA settings were as follows: 25 frames were captured with a rate of 60 frames per second. The medium cell size was 5 pixels and the minimum contrast was 80. Progressive spermatozoa have a cut off value of 50 μm/s for VAP and 80% for progressive straightness. Cells were recorded as static with a VAP cutoff < 5 μm/s, slow with a VAP cutoff 10 μm/s and medium with a VAP cutoff 50 μm/s. Furthermore, smears of spermatozoa corresponding to the three time points (0 min, 60 min, and 240 min) and the three groups were stained with eosin Y-nigrosin. Two hundred spermatozoa per slide were examined microscopically (×1000) to evaluate viability (NiconEclipse C1, Nikon, Tokyo, Japan). 

### 2.3. Quantification of the Superoxide Anion Production (Nitroblue Tetrazolium Test-NBT)

The sperm pool was centrifuged with two gradients (45% and 80%) of Percoll to remove the cryoprotectants. After centrifugation (380× *g*, 25 min, RT), the supernatant was discarded and the pellet was resuspended with 2 mL of Sperm TALP to be centrifuged twice (140× *g*, 10 min, RT). Subsequently, spermatozoa (2 × 10^6^/reaction) were supplemented with DMSO (control) or 2 different concentrations of PT (10 μM, 25 μM) and incubated for 60 min and 240 min, respectively. After ending incubation, PT was removed via centrifugation (140× *g*, 10 min, RT). Yellow colored nitroblue-tetrazolium [(2,20-bis(4-Nitrophenyl)-5,50-diphenyl-3,30-(3,30-dimethoxy-4,40-diphenylene) ditetrazolium chloride-dilution 1:10 in PBS from a stock solution of 0.01%] was added to samples for 60 min. Following incubation, the samples were washed and centrifuged at 300× *g* for 10 min in PBS twice to remove all residual NBT solution, leaving a cell pellet containing the blue formazan crystals, which were formed due to the reduction in NBT from superoxide anion. The cells and formazan crystals were dissolved in 120 μL 2M KOH and 140 μL DMSO [33]. Optical density was determined at a wavelength of 630 nm via a 96-well microplate photometer (BioTek EL800, Thomas Scientific,, Swedesboro, NJ, USA). Data were expressed as percentage of the control which was set to 100%.

### 2.4. Capacitation and Acrosome Reaction

The sperm sample was prepared as described in Section 2.3. Spermatozoa were incubated in IVF TALP medium (114 mM NaCl, 3.2 mM KCl, 0.34 mM NaH_2_PO_4_, 0.5 mM CaCl_2_, 10 mM Na lactate, 10 mg/mL phenol red, 30 μΜ penicillamine, 15 μΜ hypotaurine, 1 μM epinephrine supplemented with 10.4 mM pyruvate, 50 μg/mL gentamycin and 1% in water for embryo transfer) in the presence (positive control) or absence (negative control) of 30 mg/mL heparin (capacitating factor) [34], and with two concentrations of PT (10 μM and 25 μM) at 37 °C, 5% CO_2_ for 240 min. Subsequently, spermatozoa were exposed to calcium ionophore (10 μM in DMSO) at 37 °C for 60 min to induce acrosome reaction in the capacitated spermatozoa. After ending incubation, the samples were fixed with a fixing solution (110 mM Na_2_HPO_4_, 2.5 mM NaH_2_PO_4_, 4% paraformaldehyde, pH 7.4) for 10 min at 25 °C. Sperm samples were centrifuged and washed twice using 1.5 mL of 100 mM ammonium acetate (pH 9.0). Twenty-five μL of the sperm pellet were smeared on a glass slide and air-dried. The slides were stained with a Coomasie Blue solution (0.22% Coomassie Blue G-250, 50% methanol, 10% glacial acetic acid and 40% distilled water) for 2 min, washed thoroughly with distilled water to remove excess stain and air-dried [35]. Spermatozoa were evaluated with microscopic examination (1000×) (NiconEclipse C1, Nikon, Tokyo, Japan). Two hundred spermatozoa were evaluated as acrosome reacted or not, and the results were expressed as % of total spermatozoa.

### 2.5. Statistical Analysis

The data are presented as mean values ± SD. Statistical analysis was carried out using SPSS (version 22.0, provided by the Aristotle University of Thessaloniki). Repeated measures ANOVA with the Bonferroni correction were applied for the statistical analysis, where the interaction between different concentrations of PT and the two time points was analyzed. A value of *p* < 0.05 was considered statistically significant.

## 3. Results

### 3.1. Motility and Viability Assessment

**Figure 1:**
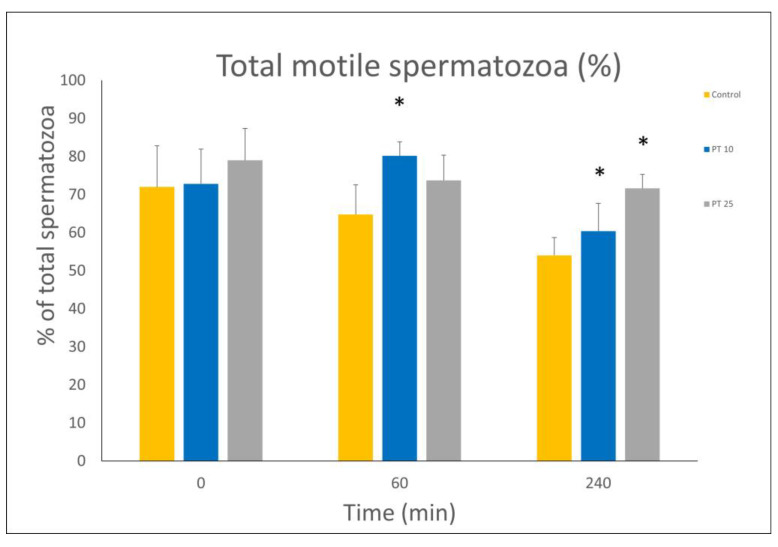
The effect of PT on the percentage of total motile spermatozoa during a 240 min incubation. Data are presented as mean ± SD. Asterisks (*) indicate statistically significant differences between the different concentrations of PT and the control group in each given time point (*p* < 0.05, *n* = 6).

The percentage of total motile spermatozoa after thawing was >70% (Figure 1). The results obtained from CASA showed a statistically significant (*p* < 0.001) decrease in the percentage of total motile spermatozoa during the 4 h incubation period for the vehicle control, while the addition of PT resulted in a time-dependent effect (*p* = 0.002) on the percentage of total motile spermatozoa (Figure 1, Table 1). Specifically, the treatment of spermatozoa with 10 μM PT maintained the percentage of total motile spermatozoa after 60 min and 240 min of incubation in statistically significant higher levels, and 25 μM PT showed similar effect on the percentage of total motile spermatozoa after 240 min of incubation (Figure 1, Table 1). PT significantly improved motility of spermatozoa, which was also accompanied by a decreased percentage of static spermatozoa after 240 min of incubation (Table 1) (*p* < 0.05).

**Figure 2:**
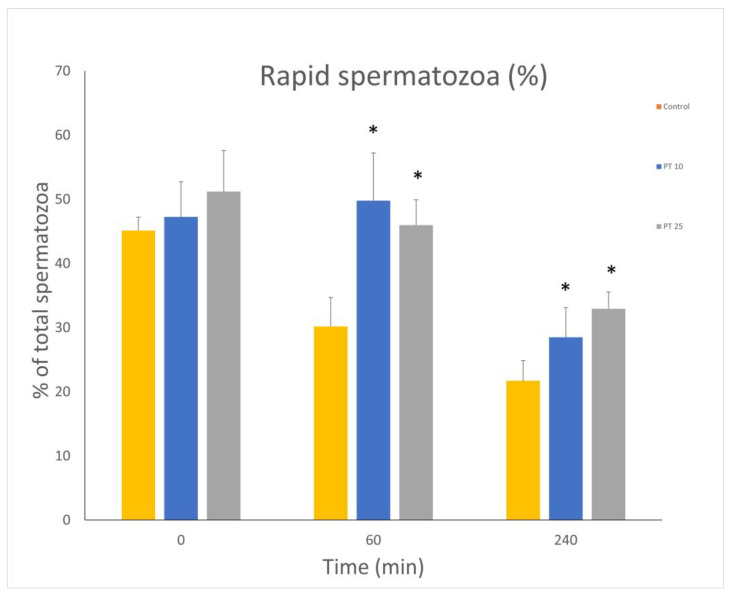
The effect of PT on the percentage of rapid spermatozoa during a 240 min incubation. Data are presented as mean ± SD. Asterisks (*) indicate statistically significant differences between the different concentrations PT and control groups in each given time point (*p* < 0.05, *n* = 6).

**Figure 3:**
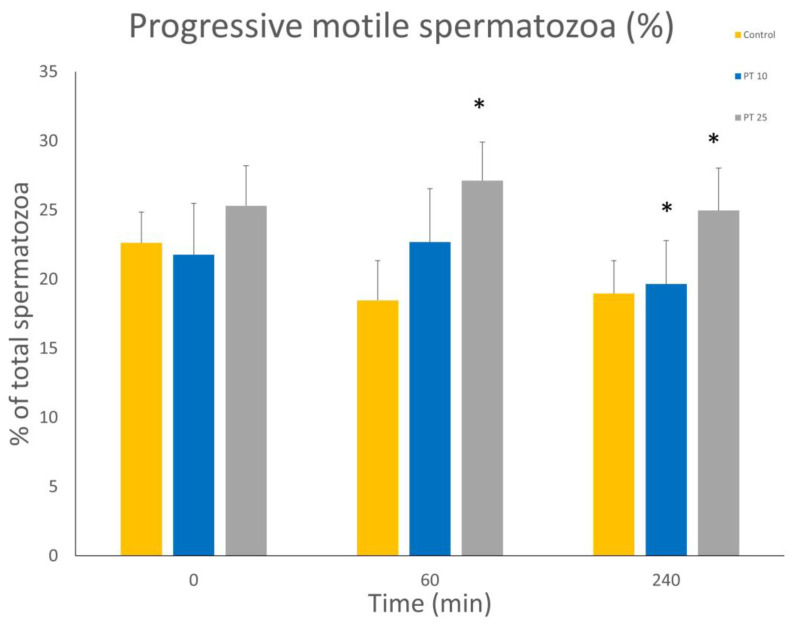
The effect of PT on the percentage of progressive motile spermatozoa during a 240 min incubation. Data are presented as mean ± SD. Asterisks (*) indicate statistically significant differences between the different concentrations PT and control groups in each given time point (*p* < 0.05, *n* = 6).

Moreover, both concentrations of PT positively affected the percentage of rapid spermatozoa in a time-dependent manner (*p* = 0.004). The results are presented in Figure 2 and Table 1. The percentage of rapid spermatozoa of the control group reduced to 30% in 60 min and to 20% in 240 min (*p* = 0.002), while treatment of spermatozoa with either 10 μΜ or 25 μΜ ΡΤ maintained the % of rapid spermatozoa after 60 min and 240 min of incubation at higher levels compared to the control group (*p* = 0.000). In addition, PT positively affected progressive motility. Specifically, 25 μΜ PT maintained the percentage of progressively motile spermatozoa after 60 min and 240 min of incubation. Moreover, the lower concentration (10 μΜ) showed a similar effect after 60 min of incubation (Table 1, Figure 3). PT did not affect other CASA kinematic parameters (Table 2).

**Figure 4:**
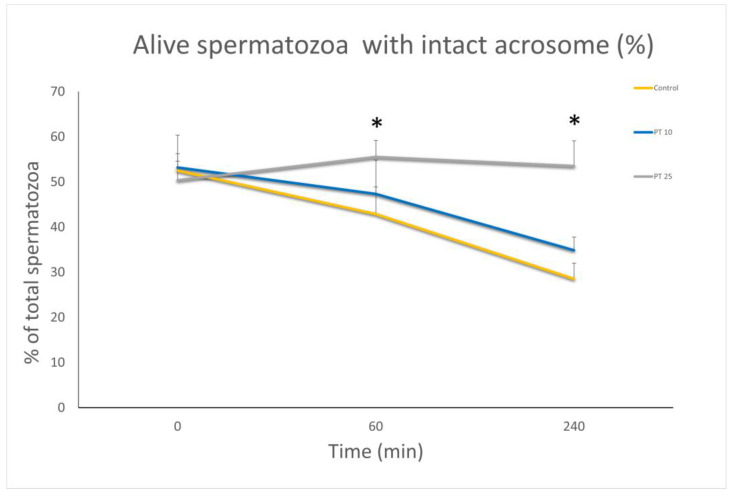
The effect of PT on the percentage of alive spermatozoa with intact acrosome during a 240 min incubation. Data are presented as mean ± SD. Asterisks (*) indicate statistically significant differences between the different concentrations PT and control groups in each time point (*p* < 0.05, *n* = 6).

The results of viability assessment are presented in Figure 4. The treatment of spermatozoa with 25 μM ΡΤ maintained the percentage of alive spermatozoa with intact acrosome during the 240 min incubation (50.23 ± 4.22%, 55.5 ± 3.67%, 53.5 ± 5.57%, for the time points 0 min, 60 min, and 240 min, respectively), showing statistically significant differences compared to the control group (*p* < 0.001). Moreover, the higher concentration of PT (25 μM) is substantially more effective in maintaining the spermatozoa alive with intact acrosome compared to the lower concentration of PT (10 μM) at 240 min of incubation (*p* = 0.007).

### 3.2. Superoxide Anion Quantification

**Figure 5:**
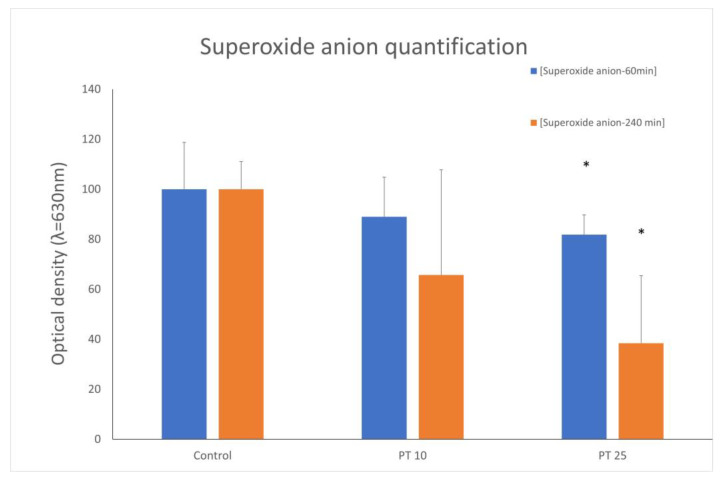
Intracellular superoxide production of frozen/thawed bovine spermatozoa supplemented with two different concentrations of PT (10 μM and 25 μM).Data are presented as mean ± SD. The percentage of the vehicle control has been set to 100% and the data are expressed as % of the control group. Asterisks (*) indicate statistically significant differences between the different concentrations of PT and the control group at each given time point (*p* < 0.05, *n* = 6).

The ROS-scavenging activity of PT was confirmed with the results of the NBT test (Figure 5). According to the results, 25 μΜ ΡΤ reduces the superoxide anion concentration compared to the control group after 60 and 240 min of incubation (*p* < 0.05).

### 3.3. Capacitation and Acrosome Reaction

**Figure 6:**
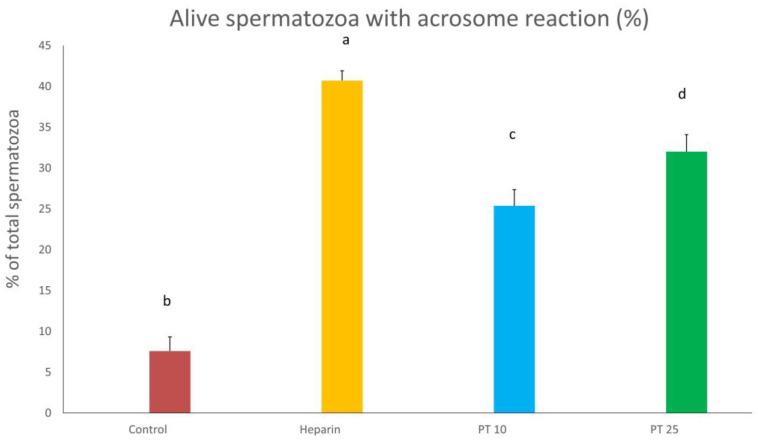
The effect of PT on the percentage of alive capacitated spermatozoa with acrosome reaction, in the presence of a positive (heparin) and a negative control Data are presented as mean ± SD. Different letters indicate statistically significant differences among groups (*p* < 0.05, *n* = 6).

Figure 6 presents the results of the Coomasie blue staining of spermatozoa. The percentage of capacitated/acrosome reacted spermatozoa in the presence of heparin, which is a well-known capacitating factor, was 40.7 ± 1.19% for the positive control, heparin. The treatment of spermatozoa with PT resulted in statistically significant differences, not only between these groups, but also with the positive and negative control (*p* < 0.05). The results obtained after microscopic evaluation were 7.58 ± 1.74%, 25.7 ± 1.97% and 32 ± 2.07%, for the control, 10 μΜ and 25 μM, respectively.

## 4. Discussion

The implication of oxidative stress in the pathophysiology of infertility and lower fertilization rates either in vivo or in vitro has been the subject of extensive research over the past 30 years [2,3,8,9]. This research also highlighted the role of antioxidants, which have been approached with enthusiasm by the scientific community and society [2,3,8,9,10]. Antioxidants have pleiotropic effects, many of which are probably still unknown. The use of natural antioxidant compounds is the new trend. The neologism “Nutraceuticals” which results from the combination of the words *Nutrition* and *Pharmaceutical*, describes ingredients or special formulations derived from food or other natural sources with health benefits.

In the present study, PT was chosen because it has a strong antioxidant activity in various cell types [25,26,27,28,29,30,31] and can be synthesized using biological and chemical methods [21]; additionally, it can be extracted from natural sources [17,18]. Moreover, the effect of PT has not yet been tested on spermatozoa of any species, including humans. The concentrations of PT tested in the present study were selected based on limited data from recent research that has mainly focused on embryos [28,32] and on our own preliminary results. Preliminary experimentation was focused on evaluation of motility, which is a critical parameter to achieve fertilization [36]. Two concentrations of PT were chosen: 10 µM and 25 µM. PT was dissolved in DMSO, which reached a final concentration in the media, not higher than 0.02%. Concentrations of DMSO < 1% are considered safe for cells and have no impact on the study of antioxidant markers, such as the concentration of ROS and the expression of antioxidant enzymes [37]. The choice of time points was based on the protocols used for sperm preparation where usually almost 1 h is required to prepare the semen before ART, while 4 h are required to capacitate bovine spermatozoa [34].

The results of our study showed that the concentration of PT which exerted an antioxidant role by eliminating the production of intracellular concentration of O_2_^−^ was the concentration of 25 µM. As mentioned before, the available literature on PT efficacy are limited and concern other cell types [25,26,27,28,29,30,31]. Therefore, any discussion could be based on the basic principles of antioxidant supplementation in ART. Furthermore, the effect of PT could be compared to that of resveratrol, despite the significant pharmacological differences that exist between the two substances, and other phenolic compounds [15,16,21]. Resveratrol is beneficial to bovine spermatozoa at concentrations ranging from 5 to 50 µM by reducing the concentration of O_2_^−^, which was also determined in this study using the NBT test [38]. Resveratrol also mediates the same effect under induced oxidative stress [39]. This phenolic compound also acts as an antioxidant with beneficial effects in murine [40] and human [41] sperm by reducing mitochondrial ROS production, scavenging ROS and/or modulating the expression of endogenous antioxidant enzymes [42]. In addition to this, a *Vitis vinifera* grape extract at a concentration of 5 μg/mL, which contains pterostilbene and other phenolic compounds, exerted an antioxidant role in thawed bull spermatozoa by suppressing the LPO levels and contributed to the maintenance of sperm quality characteristics [43]. It remains unclear to which degree this effect is attributed to PT because in this case the extract contains a multitude of antioxidant agents of the polyphenol family, including quercetin and epicatechin, which act synergistically [44].

The mitigation of O_2_^−^ is a possible explanation for the ameliorative effect of PT on bovine sperm quality characteristics. However, since PT is a lipophilic molecule, a potential effect on membrane fluidity and physiology cannot be excluded. The same mechanism has been suggested for other phenolic compounds such as quercetin [45], or lipophilic molecules such as crocetin [46]. Both concentrations of PT positively affected the percentage of total motile spermatozoa after 240 min, and thus could positively influence the fertilizing capacity of affected spermatozoa. This observation is in line with that for resveratrol [38,39]. Moreover, the antioxidant properties of PT, which were verified using the NBT test, contributed toward maintaining a higher percentage of rapid spermatozoa over time. The findings of the present study also verify that the imbalance between production and scavenging of ROS reduced the motility of spermatozoa in the control group. This effect could be attributed to the axonemal protein oxidation, mitochondrial dysfunction and/or ATP depletion [47,48]. Additionally, the induced LPO reduces motility parameters in the control group and affects the critical parameter of progressive motility [33]. In our study, PT preserved the percentage of progressive motile spermatozoa. The higher concentration was the most effective. Progressive motility is a potential predictive parameter for fertilizing capacity of bovine spermatozoa [49]. The significant protection of PT to the percentages of rapid, total, and progressive motile spermatozoa is indicative of the potential effect on the outcome of artificial insemination (AI) or in vitro fertilization [50].

The lack of antioxidant protection induces apoptotic mechanisms mediated by the activation of caspases and the release of cytochrome c from mitochondria, which lead cells to death [51]. The implication of these findings may explain the downward trend in terms of motility and viability parameters presented by the control group, while the concentration of 25 µM PT positively affected the motility parameters during the 240 min incubation. The low concentration of PT (10 μΜ) also exerted a positive effect on motility parameters, without any effect on the levels of O_2_^−^ and viability. Considering that we quantified only O_2_^−^, the measurement of the concentration of other ROS (e.g., H_2_O_2_) and LPO to further elucidate the effect of PT on the oxidative balance of spermatozoa and consequently on sperm parameters, a safer conclusion could be drawn. Interestingly, PT had no effect on other CASA kinematic parameters (VCL, VSL, VAP, and ALH). One possible explanation is that these parameters could be affected by concentrations of PT other than those used in the present study. Moreover, some antioxidants may positively affect spermatozoa in terms of DNA integrity; however, it cannot be overlooked that they adversely affect or do not affect the motility parameters. Similar results have been reported in another study on human spermatozoa where α-tocopherol did not affect the motility parameters; α-tocopherol is known as a strong antioxidant agent for spermatozoa [52].

Published data indicate that significant amounts of ROS are produced during thawing of bovine spermatozoa, which are responsible for the production of lipid hydroperoxides and loss of cell membrane fluidity and integrity [5]. During experimentation, spermatozoa were subjected to intense stress, not only due to freeze/thaw and centrifugations [4,5], but also due to the prolonged incubation at 37 °C under conditions of high oxygen tension, compared to that of the genital tract (21% vs. 5%) [7]. The results of the present study revealed that the addition of 25 µM PT creates a stable environment for spermatozoa by scavenging O_2_^−^. Furthermore, the beneficial role of PT on sperm viability could be exerted through estrogen receptor beta (ERβ). This effect has been verified in other cell types, such as myoblasts where PT binds to ERβ and protects cells through the induction of SOD production without affecting mitochondrial ROS production [30,31]. Finally, the presence of PT in the lipid phase of the cell membrane would probably protect the cells from the initiation of LPO. The last mechanism has been proposed for other phenolic compounds, such as quercetin [53].

Finally, PT has been tested under capacitating conditions. Capacitation is a prerequisite for fertilization [54] and includes a series of biochemical and molecular events in which ROS seem to play a central role [14]. Controlled amounts of ROS are required for protein tyrosine phosphorylation and activation of both human and bovine spermatozoa [4,12,13,14]. Superoxide anion plays a central role in the process of capacitation in bovine spermatozoa [3,4,12]. As a result, the quantification of O_2_^−^ concentration in the present study could be correlated to the percentages of capacitated/acrosome-reacted spermatozoa. Treatment of spermatozoa treated with 25 µM PT resulted in higher percentages of capacitated/acrosome-reacted spermatozoa compared to the negative control group. The effect of PT on capacitation processes can be attributed to the regulation of O_2_^−^ production and LPO to levels required for these processes. It is noteworthy that even the lower concentration of PT (10 µM) affected the percentage of capacitated/acrosome reacted spermatozoa. Moreover, a more direct effect of PT cannot be excluded due to its lipophilic properties. It is possible that PT increases the permeability to calcium ions and bicarbonate ions, which lead to the metabolic pathways to initiate sperm activation [55]. Taking under consideration that 25 μΜ PT positively affects the progressive motility and induces capacitation/acrosome reaction, we could postulate that PT regulates the functions of specific transmembrane enzymes and calcium ions related to the acquisition of fertilizing capacity. 

The beneficial role of PT on the fertilizing capacity of bovine spermatozoa is indisputable, and the latter has been verified in in vitro embryo production. PT at a concentration of 0.25 µM significantly improved embryonic development on the 4th day of culture in mice, and this action was exerted through the enhancement of antioxidant and antiapoptotic mechanisms. On the contrary, a study conducted in bovine embryos showed that the addition of pterostilbene at concentrations ranging from 0.11 µM to 3 µM significantly reduced the percentage of blastocysts produced on day 7 of in vitro culture, which was attributed to depriving the embryos of the required amounts of ROS for embryonic development [31]. New studies could be addressed to further clarify the role of PT in sperm physiology.

## 5. Conclusions

In this paper, we investigated the role of PT on sperm physiology. This role seems to be very promising for the optimization of sperm quality characteristics. The data of the present study revealed that PT maintained the percentage of total motile, rapidly moving, and alive spermatozoa. This effect was exerted through the elimination of superoxide anion production compared to the control group. Moreover, PT mediated capacitation. This effect could be attributed to the modulation of superoxide anion. However, a more direct effect cannot be disregarded because the molecule is lipophilic, which implies that it could affect the membrane physiology. Further studies should be carried out to clarify the mechanism of action, the safety and efficacy of PT on in vitro fertilization in bovine species. The effect of PT could be evaluated in terms of the number and quality of the produced blastocysts. Finally, PT could be also tested as an additive in the freezing medium in order to protect spermatozoa from the damage mainly inflicted by ROS overproduction during freeze/thawing.

## Figures and Tables

**Table 1:** The effect of different concentrations of PT (0, 10, and 25 μM) on motility parameters of frozen/thawed bovine spermatozoa during 240 min of incubation. Data are presented as mean ± SD. Asterisks (*) indicate statistically significant differences between the different concentrations of PT and the control group in each given time point (*p* < 0.05, *n* = 6).

Time Point	Group	Rapid (%)	Medium (%)	Slow (%)	Total Motile (%)	Static (%)	Progressive Motile (%)
0 min	Control	45.13 ± 2.07	22.63 ± 8.70	4.21 ± 2.86	71.98 ± 10.81	29.88 ± 8.44	22.61 ± 2.24
	ΡT 10	47.25 ± 5.45	20.13 ± 3.09	5.42 ± 3.10	72.80 ± 9.13	27.18 ± 9.11	21.77 ± 3.71
	ΡT 25	51.2 ± 6.37	23.26 ± 5.63	4.53 ± 1.71	79 ± 8.31	21 ± 8.31	25.31 ± 2.88
60 min	Control	30.18 ± 4.51	24.83 ± 5.43	9.73 ± 3.78	64.75 ± 7.79	33.6 ± 9.1	18.46 ± 2.87
	ΡT 10	49.8 ± 7.43 *	20.95 ± 10.39	9.4 ± 7.89	80.15 ± 3.7 *	19.86 ± 3.72 *	22.68 ± 3.85
	ΡT 25	45.96 ± 3.95 *	24.26 ± 4.56	3.45 ± 1.80	73.68 ± 6.61	26.31 ± 6.61	27.11 ± 2.79 *
240 min	Control	21.7 ± 3.16	22.44 ± 6.96	15.57 ± 6.52	54 ± 5.15	43.27 ± 8.58	18.95 ± 2.38
	ΡT 10	28.48 ± 4.64 *	24.05 ± 4.97	7.83 ± 4.15	60.36 ± 7.28 *	39.65 ± 7.28 *	19.65 ± 3.13 *
	ΡT 25	32.95 ± 3.92 *	28.05 ± 4.22	10.61 ± 3.11	71.61 ± 3.69 *	28.73 ± 3.73 *	24.96 ± 3.06 *

**Table 2:** The effect of different concentrations of PT (0, 10 μΜ, and 25 μΜ) on CASA kinematic parameters of frozen/thawed bovine spermatozoa during 240 min of incubation. Data are presented as mean ± SD (*p* < 0.05, *n* = 6).

Time Point	Group	VCL (μm/s)	VSL (μm/s)	VAP (μm/s)	ALH
0 min	Control	62.43 ± 6.92	20.06 ± 2.29	34.41 ± 2.16	3.26 ± 0.62
	ΡT 10	58.03 ± 9.27	18.67 ± 2.05	30.73 ± 4.8	3.03 ± 0.28
	ΡT 25	65.2 ± 9.39	20.03 ± 1.36	36.13 ± 1.87	3.27 ± 0.52
60 min	Control	54 ± 2.53	20.02 ± 3.67	32.75 ± 3.2	2.98 ± 0.29
	ΡT 10	59.48 ± 6.36	25.33 ± 7.72	37.7 ± 6.09	2.76 ± 0.28
	ΡT 25	62.51 ± 4.64	24.9 ± 2.09	38.66 ± 2.85	2.95 ± 0.34
240 min	Control	53.75 ± 10.51	24.78 ± 7.73	34.92 ± 7.1	2.42 ± 0.23
	ΡT 10	48.95 ± 11.46	19.88 ± 7.55	31.55 ± 9.4	2.88 ± 0.47
	ΡT 25	51.35 ± 10.99	21.31 ± 7.39	32.98 ± 7.79	3.01 ± 0.42

VCL: curvillinear velocity, VSL: straight line velocity, VAP: average path velocity, ALH: amplitude lateral head.

## Data Availability

The data presented in this study are available on request from the corresponding authors.

## References

[1] Chaudière J., Rice-Evans C.A., Burdon R.H. (1994). Some chemical and biochemical constraints of oxidative stress in living cells. New Comprehensive Biochemistry, Free Radical Damage and Its Control.

[2] Sanocka D., Kurpisz M. (2004). Reactive oxygen species and sperm cells. Reprod. Biol. Endocrinol..

[3] Sikka S.C., Rajasekaran M., Hellstrom W.J. (1995). Role of oxidative stress and antioxidants in male infertility. J. Androl..

[4] Aitken R.J., Clarkson J.S. (1998). Significance of reactive oxygen species and antioxidants in defining the efficacy of sperm preparation techniques. J. Androl..

[5] Chatterjee S., Gagnon C. (2001). Production of reactive oxygen species by spermatozoa undergoing cooling, freezing, and thawing. Mol. Reprod. Dev..

[6] Correa J.R., Pace M.M., Zavos P.M. (1997). Relationships among frozen-thawed sperm characteristics assessed via the routine semen analysis, sperm functional tests and fertility of bulls in an artificial insemination program. Theriogenology.

[7] Hoshi H. (2003). In vitro production of bovine embryos and their application for embryo transfer. Theriogenology.

[8] Al-Gubory K.H., Fowler P.A., Garrel C. (2010). The roles of cellular reactive oxygen species, oxidative stress and antioxidants in pregnancy outcomes. Int. J. Biochem. Cell Biol..

[9] Agarwal A., Durairajanayagam D., du Plessis S.S. (2010). Utility of antioxidants during assisted reproductive techniques: An evidence based review. Reprod. Biol. Endocrinol..

[10] Pintus E., Ros-Santaella J.L. (2021). Impact of Oxidative Stress on Male Reproduction in Domestic and Wild Animals. Antioxidants.

[11] Sapanidou V., Tsantarliotou M.P., Lavrentiadou S.N. (2023). A review of the use of antioxidants in bovine sperm preparation protocols. Anim. Reprod. Sci..

[12] de Lamirande E., Gagnon C. (1993). A positive role for the superoxide anion in triggering hyperactivation and capacitation of human spermatozoa. Int. J. Androl..

[13] O’Flaherty C., Beorlegui N., Beconi M.T. (2003). Participation of superoxide anion in the capacitation of cryopreserved bovine sperm. Int. J. Androl..

[14] Visconti P.E. (2009). Understanding the molecular basis of sperm capacitation through kinase design. Proc. Natl. Acad. Sci. USA.

[15] Beecher G.R. (2003). Overview of dietary flavonoids: Nomenclature, occurrence and intake. J. Nutr..

[16] Rahman M.M., Rahaman M.S., Islam M.R., Rahman F., Mithi F.M., Alqahtani T., Almikhlafi M.A., Alghamdi S.Q., Alruwaili A.S., Hossain M.S. (2021). Role of Phenolic Compounds in Human Disease: Current Knowledge and Future Prospects. Molecules.

[17] Manach C., Scalbert A., Morand C., Rémésy C., Jiménez L. (2004). Polyphenols: Food sources and bioavailability. Am. J. Clin. Nutr..

[18] Pratt D.E., Hudson B.J.F., Hudson B.J.F. (1990). Natural Antioxidants Not Exploited Commercially. Food Antioxidants.

[19] Sobolev V.S., Khan S.I., Tabanca N., Wedge D.E., Manly S.P., Cutler S.J., Coy M.R., Becnel J.J., Neff S.A., Gloer J.B. (2011). Biological activity of peanut (*Arachis hypogaea*) phytoalexins and selected natural and synthetic Stilbenoids. J. Agric. Food Chem..

[20] Liu Y., You Y., Lu J., Chen X., Yang Z. (2020). Recent Advances in Synthesis, Bioactivity, and Pharmacokinetics of Pterostilbene, an Important Analog of Resveratrol. Molecules.

[21] Kapetanovic I.M., Muzzio M., Huang Z., Thompson T.N., McCormick D.L. (2011). Pharmacokinetics, oral bioavailability, and metabolic profile of resveratrol and its dimethylether analog, pterostilbene, in rats. Cancer Chemother. Pharmacol..

[22] Chen Z.P., Cai Y., Phillipson J.D. (1994). Studies on the anti-tumor, anti-bacterial, and wound-healing properties of dragon’s blood. Planta Medica.

[23] Remsberg C.M., Yáñez J.A., Ohgami Y., Vega-Villa K.R., Rimando A.M., Davies N.M. (2008). Pharmacometrics of pterostilbene: Preclinical pharmacokinetics and metabolism, anticancer, antiinflammatory, antioxidant and analgesic activity. Phytother. Res..

[24] Gupta R., Gupta R.S. (2011). Effect of Pterocarpus marsupium on Streptozotocin-induced Oxidative Stress in Kidney of Diabetic Wistar Rats. J. Herbs Spices Med..

[25] Messina F., Guglielmini G., Curini M., Orsini S., Gresele P., Marcotullio M.C. (2015). Effect of substituted stilbenes on platelet function. Fitoterapia.

[26] Zhou J., Ci X., Ma X., Yu Q., Cui Y., Zhen Y., Li S. (2019). Pterostilbene Activates the Nrf2-Dependent Antioxidant Response to Ameliorate Arsenic-Induced Intracellular Damage and Apoptosis in Human Keratinocytes. Front. Pharmacol..

[27] Li J., Deng R., Hua X., Zhang L., Lu F., Coursey T.G., Pflugfelder S.C., Li D.Q. (2016). Blueberry Component Pterostilbene Protects Corneal Epithelial Cells from Inflammation via Anti-oxidative Pathway. Sci. Rep..

[28] Sosa F., Romo S., Kjelland M.E., Álvarez-Gallardo H., Pérez-Reynozo S., Urbán-Duarte D., De La Torre-Sánchez J.F. (2020). Effect of pterostilbene on development, equatorial lipid accumulation and reactive oxygen species production of in vitro-produced bovine embryos. Reprod. Domest. Anim..

[29] Amarnath Satheesh M., Pari L. (2006). The antioxidant role of pterostilbene in streptozotocin-nicotinamide-induced type 2 diabetes mellitus in Wistar rats. J. Pharm. Pharmacol..

[30] McCormack D., McFadden D. (2013). A review of pterostilbene antioxidant activity and disease modification. Oxidative Med. Cell. Longev..

[31] Robb E.L., Stuart J.A. (2014). The stilbenes resveratrol, pterostilbene and piceid affect growth and stress resistance in mammalian cells via a mechanism requiring estrogen receptor beta and the induction of Mn-superoxide dismutase. Phytochemistry.

[32] Ullah O., Li Z., Ali I., Xu L., Liu H., Jin H.Z., Fang Y.Y., Jin Q.G., Fang N. (2019). Pterostilbene exerts a protective effect via regulating tunicamycin-induced endoplasmic reticulum stress in mouse preimplantation embryos. Vitr. Cell. Dev. Biol. Anim..

[33] Becerra M.C., Eraso A.J., Albesa I. (2003). Comparison of oxidative stress induced by ciprofloxacin and pyoverdin in bacteria and in leukocytes to evaluate toxicity. J. Lumin..

[34] Parrish J.J., Susko-Parrish J., Winer M.A., First N.L. (1988). Capacitation of bovine sperm by heparin. Biol. Reprod..

[35] Larson J.L., Miller D.J. (1999). Simple histochemical stain for acrosomes on sperm from several species. Mol. Reprod. Dev..

[36] Kasimanickam R., Pelzer K.D., Kasimanickam V., Swecker W.S., Thatcher C.D. (2006). Association of classical semen parameters, sperm DNA fragmentation index, lipid peroxidation and antioxidant enzymatic activity of semen in ram-lambs. Theriogenology.

[37] Tamagawa S., Sakai D., Schol J., Sako K., Nakamura Y., Matsushita E., Warita T., Hazuki S., Nojiri H., Sato M. (2020). N-acetylcysteine attenuates oxidative stress-mediated cell viability loss induced by dimethyl sulfoxide in cryopreservation of human nucleus pulposus cells: A potential solution for mass production. JOR Spine.

[38] Tvrdá E., Lukáč N., Lukáčova J., Hashim F., Massányi P. (2015). In vitro supplementation of resveratrol to bovine spermatozoa: Effects on motility, viability and superoxide production. J. Microbiol. Biotechnol. Food Sci..

[39] Tvrdá E., Kováčik A., Tušimová E., Massányi P., Lukáč N. (2015). Resveratrol offers protection to oxidative stress induced by ferrous ascorbate in bovine spermatozoa. J. Environ. Sci. Health Part A.

[40] Mojica-Villegas M.A., Izquierdo-Vega J.A., Chamorro-Cevallos G., Sánchez-Gutiérrez M. (2014). Protective Effect of Resveratrol on Biomarkers of Oxidative Stress Induced by Iron/Ascorbate in Mouse Spermatozoa. Nutrients.

[41] Collodel G., Federico M.G., Geminiani M., Martini S., Bonechi C., Rossi C., Figura N., Moretti E. (2011). Effect of trans-resveratrol on induced oxidative stress in human sperm and in rat germinal cells. Reprod. Τoxicol..

[42] Pervaiz S., Holme A.L. (2009). Resveratrol: Its biologic targets and functional activity. Antioxid. Redox Signal..

[43] Sapanidou V.G., Margaritis I., Siahos N., Arsenopoulos K., Dragatidou E., Taitzoglou I.A., Zervos I.A., Theodoridis A., Tsantarliotou M.P. (2014). Antioxidant effect of a polyphenol-rich grape pomace extract on motility, viability and lipid peroxidation of thawed bovine spermatozoa. J. Biol. Res..

[44] Stagos D., Kazantzoglou G., Theofanidou D., Kakalopoulou G., Magiatis P., Mitaku S., Kouretas D. (2006). Activity of grape extracts from Greek varieties of *Vitis vinifera* against mutagenicity induced by bleomycin and hydrogen peroxide in *Salmonella typhimurium* strain TA102. Mutat. Res..

[45] Ben Abdallah F., Zribi N., Ammar-Keskes L. (2011). Antioxidative potential of Quercetin against hydrogen peroxide induced oxidative stress in spermatozoa in vitro. Andrologia.

[46] Sapanidou V., Taitzoglou I., Tsakmakidis I., Kourtzelis I., Fletouris D., Theodoridis A., Lavrentiadou S., Tsantarliotou M. (2016). Protective effect of crocetin on bovine spermatozoa against oxidative stress during in vitro fertilization. Andrology.

[47] de Lamirande E., Gagnon C. (1992). Reactive oxygen species and human spermatozoa. I. Effects on the motility of intact spermatozoa and on sperm axonemes. J. Androl..

[48] Aitken R.J., Harkiss D., Buckingham D. (1993). Relationship between iron-catalysed lipid peroxidation potential and human sperm function. J. Reprod. Fertil..

[49] Li Y., Kalo D., Zeron Y., Roth Z. (2016). Progressive motility—A potential predictive parameter for semen fertilization capacity in bovines. Zygote.

[50] Kathivaran P., Kalatharan J., Karthikeya G., Rengarajan K., Kadirvel G. (2011). Objective sperm analysis to assess dairy bull fertility using computer-aided system: A review. Reprod. Domest. Anim..

[51] Aitken R.J., Koppers A.J. (2011). Apoptosis and DNA damage in human spermatozoa. Asian J. Androl..

[52] Donnelly E.T., McClure N., Lewis S.E. (1999). The effect of ascorbate and alpha-tocopherol supplementation in vitro on DNA integrity and hydrogen peroxide-induced DNA damage in human spermatozoa. Mutagenesis.

[53] Tvrdá E., Tušimová E., Kováčik A., Paál D., Libová Ľ., Lukáč N. (2016). Protective Effects of Quercetin on Selected Oxidative Biomarkers in Bovine Spermatozoa Subjected to Ferrous Ascorbate. Reprod. Domest. Anim..

[54] Baker M.A., Weinberg A., Hetherington L., Villaverde A.I., Velkov T., Baell J., Gordon C.P. (2015). Defining the mechanisms by which the reactive oxygen species by-product, 4-hydroxynonenal, affects human sperm cell function. Biol. Reprod..

[55] Aitken R.J., Nixon B. (2013). Sperm capacitation: A distant landscape glimpsed but unexplored. Mol. Human Reprod..

